# Comparative Assessment of Pharyngeal Airway Dimensions in Skeletal Class I, II, and III Emirati Subjects: A Cone Beam Computed Tomography Study

**DOI:** 10.3390/dj12100301

**Published:** 2024-09-25

**Authors:** Sara AlAskar, Mohamed Jamal, Amar Hassan Khamis, Ahmed Ghoneima

**Affiliations:** 1 Hamdan Bin Mohammed College of Dental Medicine (HBMCDM), Mohammed Bin Rashid University of Medicine and Health Sciences, Dubai P.O. Box 505055, United Arab Emirates; 2 Indiana University School of Dentistry, Indianapolis, IN 46202, USA

**Keywords:** cone beam computed tomography, airway volume, CBCT

## Abstract

The aim of the current study was to evaluate the pharyngeal airway dimensions of individuals with different skeletal patterns in a cohort of the Emirati population. The specific aim was to assess the relationship between pharyngeal airway dimensions and anterior facial height in relation to different skeletal patterns. This retrospective study was conducted on a sample of 103 CBCT scans of adult Emirati subjects categorized into three groups according to their skeletal classification as indicated by the ANB angle: Class I (n = 35), Class II (n = 46), and Class III (n = 22). All CBCT scans were taken using an i-CAT CBCT imaging machine (Imaging Sciences, Hatfield, PA, USA). The age range of the patients was 19 to 68 years (62 women and 41 men). ANOVA, *t*-tests, Kruskal–Wallis, and Mann–Whitney tests were employed for comparing means among groups. The correlation coefficient was used to evaluate the association between variables. A *p*-value of less than 0.05 was considered statistically significant. This study revealed significant associations between various airway parameters and cephalometric measurements. Positive correlations were observed between nasal cavity volume and nasopharynx volume, as well as anterior facial height. Oropharynx volume exhibited positive correlations with hypopharynx volume and total airway volume, and negative correlations with overjet, ANB angle, and patient age. Hypopharynx volume correlated positively with total airway volume and the most constricted area of the airway (MCA). Total airway volume showed positive correlations with MCA and anterior facial height. MCA had negative correlations with ANB angle and patient age. Nasopharynx volume was significantly larger in the skeletal Class I group than in the Class II or Class III groups, while the other airway parameters showed no significant differences among the groups (*p* > 0.05). Several airway parameters showed a correlation with anterior facial height among the different skeletal patterns. Nasopharyngeal airway volume was significantly larger in the skeletal Class I group than in Class II and III groups in the studied sample.

## 1. Introduction

Airway volume and respiratory functions are highly relevant to the orthodontic specialty. Disruptions in normal respiratory patterns during active craniofacial development can result in significant craniofacial abnormalities. Studies have highlighted associations between airway-related disorders, such as obstructive sleep apnea (OSA), and craniofacial anomalies, such as retrognathia or narrow palate. This bidirectional relationship underscores the importance of considering both airway function and craniofacial morphology in orthodontic diagnosis and treatment planning [1,2,3].

Malocclusion is defined as an abnormal relationship of dental arches, with or without irregularities of the teeth. It is considered a developmental disorder and a public dental health problem with high prevalence and treatment needs. Altered oral functions such as mastication, speech, and swallowing may lead to orofacial adaptability, resulting in malocclusion. Studies have confirmed that airway problems are significantly related to different types of malocclusions and that nasal obstruction is a major etiological factor for dentofacial anomalies [4,5,6].

The anatomy and function of the pharyngeal airway play a pivotal role in craniofacial growth and development. As the airway is a dynamic conduit for airflow during respiration, any disturbances in the form or function of the airway can profoundly affect craniofacial morphology. For instance, chronic mouth breathing, often associated with airway obstruction, can alter facial muscle activity and lead to malocclusion and skeletal discrepancies [6,7,8]. The prevalence of malocclusion varies among different ethnic groups, age groups, and sexes [9]. The current study aimed to evaluate the pharyngeal airway dimensions of individuals with different skeletal patterns in a cohort of the Emirati population. The specific aim was to assess the relationship between pharyngeal airway dimensions and anterior facial height in relation to different skeletal patterns.

## 2. Materials and Methods

This retrospective study was conducted on pre-treatment craniofacial cone beam computed tomography (CBCT) scans of 103 adult Emirati subjects with different skeletal classifications obtained from the archives of Dubai Dental Hospital, Dubai, UAE. All CBCT scans were taken during the period of 2019–2023 using an i-CAT CBCT machine (Imaging Sciences, Hatfield, PA, USA) with a standard setting of a full 13 cm field of view, 20 s scanning time, and a 0.4 mm voxel size resolution. The CBCT scans were de-identified and coded and the study was approved by the Institutional Review Board of Mohammed Bin Rashid University of Medicine and Health Sciences. The sample consisted of 62 females and 41 males (age range of 19 to 68 years). Subjects were categorized into three groups according to their skeletal classification as indicated by the ANB angle (A point, nasion, B point) (Class I: n = 35; Class II: n = 46; and Class III: n = 22). This study was approved by the Institutional Review Board of Mohammed Bin Rashid University of Medicine and Health Sciences, MBRU IRB-2023-375.

**Figure 1:**
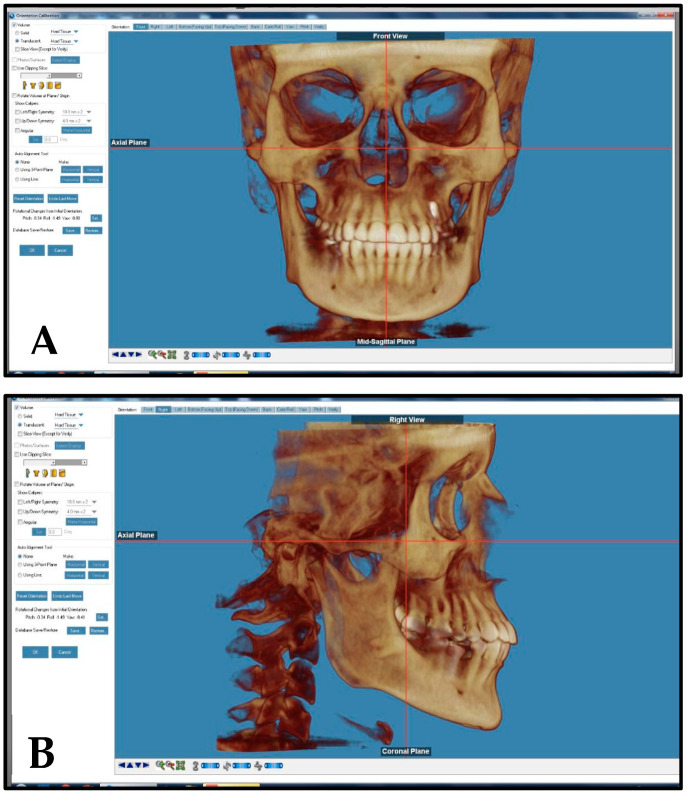
(**A**) Midsagittal plane adjusted on the skeletal midline, (**B**) the axial plane adjusted to show the Frankfort horizontal plane (right porion to right orbitale), and coronal plane adjusted as a tangent to the posterior border of the zygomatic process.

**Figure 2:**
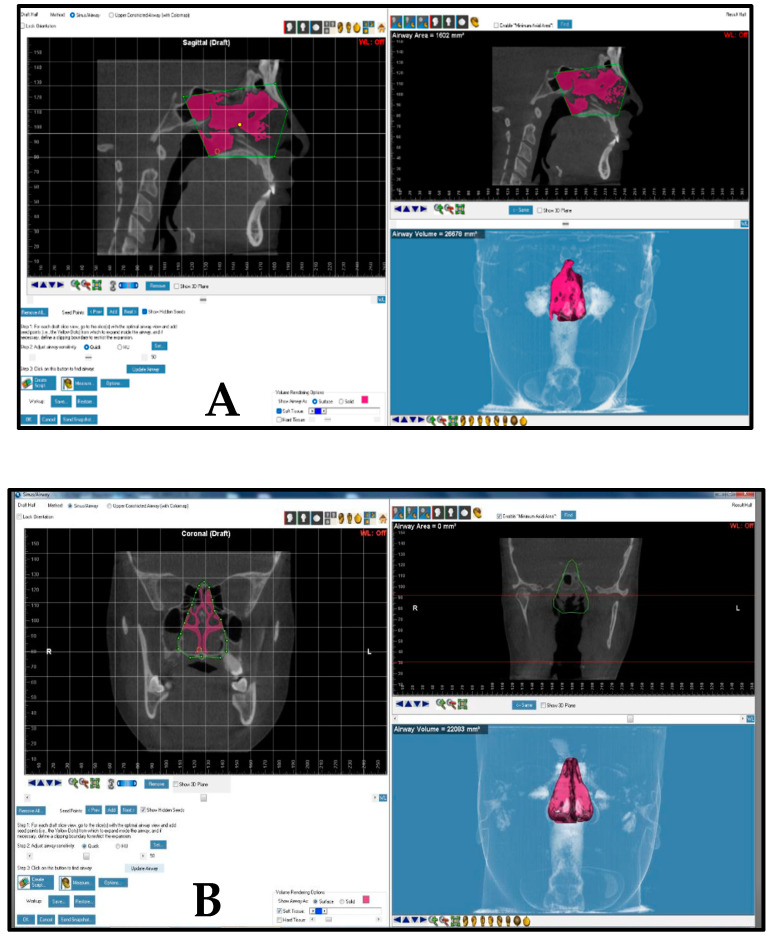
Nasal cavity boundaries and volume. The boundaries of the nasal cavity included lines from ANS to PNS to tip of nasal process to sella in (**A**) sagittal view and (**B**) coronal view.

**Figure 3:**
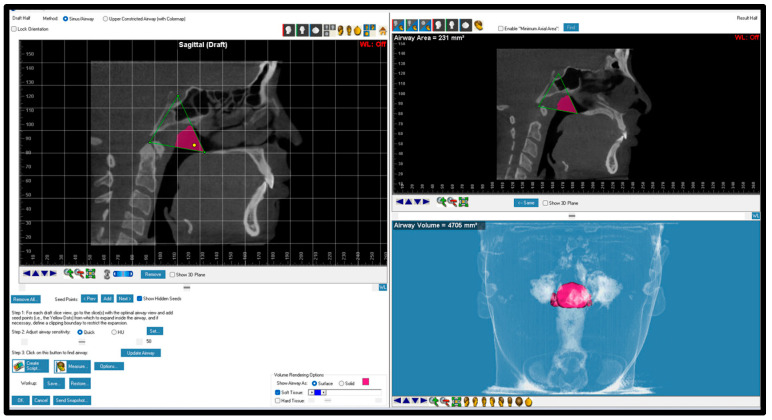
Nasopharynx boundaries and volume. The boundaries of the nasopharynx included lines from posterior nasal spine to sella to superior part of odontoid process.

**Figure 4:**
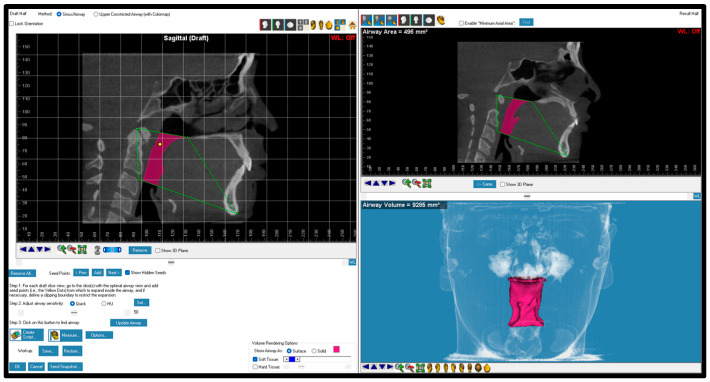
Oropharynx boundaries and volume. The boundaries of the oropharynx included lines from posterior nasal spine to superior part of odontoid process to most superior part of CV3 to menton. CV: cervical vertebrae.

**Figure 5:**
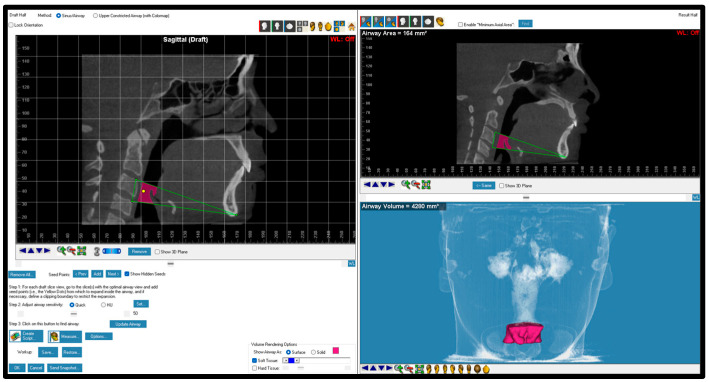
Hypopharynx boundaries and volume. The boundaries of the hypopharynx included lines from menton to most anterior superior part of CV3 to most anterior superior part of CV4.

**Figure 6:**
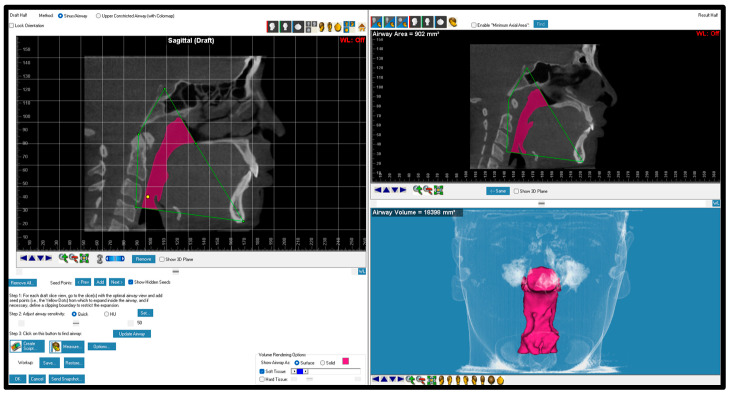
Total airway boundaries and volume. The boundaries of the total airway included lines from posterior nasal spine to sella to superior part of odontoid process to most anterior superior part of CV4 to menton.

**Figure 7:**
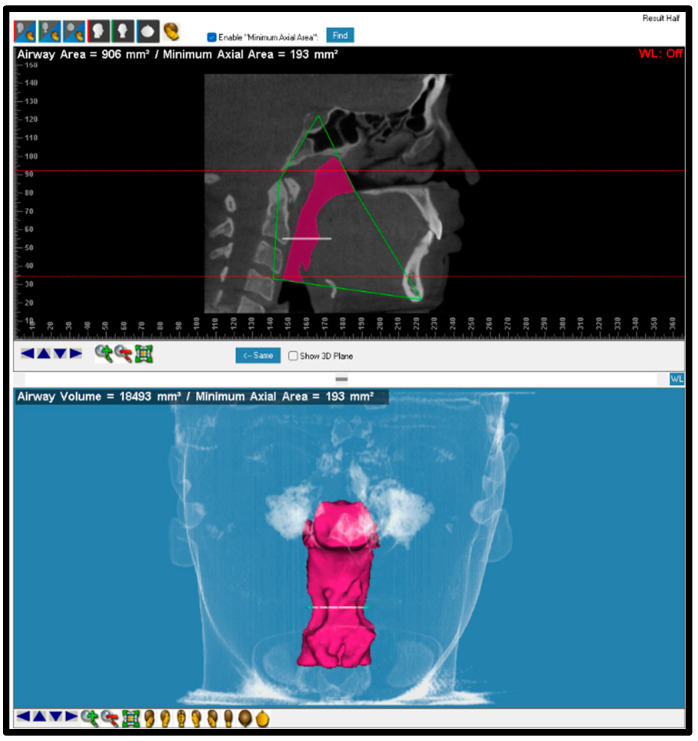
Most constricted area was detected by software after drawing two lines. The first line represents the superior part of the airway, and the second line represents the inferior part of the airway.

CBCT scans that had poor quality, missing anatomical information, cloudiness in the airway, mixed dentition stage, previous orthodontic treatment, asymmetrical skeletal pattern, orthognathic surgery, and/or craniofacial anomalies were excluded from this study. Expatriate residents and non-Emirati individuals were also excluded from this study. Before landmark identification, the 3-dimensional (3D) volumetric images were oriented with Dolphin imaging software (version 11.95, Dolphin Imaging, Chatsworth, CA, USA) as follows: the midsagittal plane was adjusted on the skeletal midline of the face, the axial plane was adjusted to show the Frankfort horizontal plane (right porion to right orbitale), and the coronal plane was adjusted to pass through the posterior border of the zygomatic process (Figure 1). Pharyngeal airway areas identified and measured in this study included the nasal cavity, nasopharynx, oropharynx, hypopharynx, total airway, and the most constricted airway area (MCA). The definitions of the selected airway parameters and cephalometric measurements are presented in Table 1 and Figure 2, Figure 3, Figure 4, Figure 5, Figure 6 and Figure 7. The boundaries of each airway segment and the MCA are defined in Table 2.

Inter-rater reliability measures were conducted by the primary investigator (S.A.) at the start of this study using 10 randomly selected CBCT scans measured twice with a two- week period in between. Records were coded and randomized. The cephalometric parameters, airway volumes, and MCA were identified and measured using Dolphin 3D imaging software (version 11.95, Dolphin Imaging, Chatsworth, CA, USA).

### Statistical Analysis

The data were entered into a computer using IBM-SPSS for Windows, version 29.0 (SPSS Inc., Armonk, NY, USA). The total sample size of 103 subjects provided 80% power to detect a correlation coefficient of 0.3 significantly different from zero. Categorical variables were described using proportions, while continuous variables were characterized using measures of central tendency (mean and median) and measures of dispersion (standard deviation and interquartile range). The normality of continuous variables was reassessed using the Kolmogorov–Smirnov test. ANOVA and *t*-tests were employed for comparing means among more than two groups or between two groups, respectively. In cases where the data were not normally distributed or when comparing means among more than two groups, the Kruskal–Wallis test and Mann–Whitney test were utilized. The correlation coefficient was used to evaluate the association between continuous variables. A *p*-value of less than 0.05 was considered statistically significant in all analyses.

## 3. Results

The inter-rater reliability was considered excellent (all intraclass correlation coefficients (ICCs) between the two readings were ≥0.90). No statistically significant differences existed between the two readings. The descriptive statistics of the measured airway segments and cephalometric parameters are presented in Table 3.

The correlations of the different airway parameters and cephalometric parameters indicated significant associations between some parameters. At the level of the nasal cavity, positive correlations were found between the nasal cavity volume and each of the nasopharynx volume and anterior facial height (N-Me) at a significance level of 0.01. At the level of the nasopharynx, positive correlations were found between the nasopharynx volume and each of the oropharynx volume, total airway, MCA, ANB angle, and anterior facial height (N-Me) at a significance level of 0.01 (Table 4).

At the level of the oropharynx, the oropharynx volume had a positive correlation with the hypopharynx volume, total airway, and MCA at a significance level of 0.01. However, negative correlations existed between the oropharynx volume and overjet, ANB angle, and age of the patients at a significance level of 0.05. At the level of the hypopharynx, there were positive correlations between the hypopharynx volume and each of the total airway volume and MCA at a significance level of 0.01 (Table 4).

Positive correlations existed between the total airway volume and each of the MCA at a significance level of 0.01 and anterior facial height (N-Me) at a significance level of 0.05. The MCA had a negative correlation with the ANB angle at a significance level of 0.05 and with the age of the patients at a significance level of 0.01. All other cephalometric parameters showed non-significant correlations with the airway parameters (Table 4).

Nasopharynx volume was the only airway segment showing significant differences among the skeletal groups, with a larger volume in Class I than in Class II or Class III (*p* = 0.031). No significant differences were found for any other measurement, including the MCA size/location among the groups (*p* > 0.05) (Table 5). The MCA was not located at the same airway level, even within the same skeletal group (Table 6).

## 4. Discussion

Understanding craniofacial growth and development involves unraveling the intricate interplay of the genetic, environmental, and functional factors that shape the face and surrounding structures. Over the years, research in this field has evolved significantly, exploring various theories to elucidate the complex mechanisms underlying craniofacial growth. One prominent theory, Moss’s functional matrix theory, posits that the growth of the craniofacial complex is heavily influenced by the functional demands imposed on the surrounding soft tissues. According to this theory, interactions between soft tissues, such as muscles and ligaments, and the underlying skeletal framework dynamically shape facial morphology [10,11,12]. The transition from nasal to oronasal breathing patterns triggers functional adaptations that have been linked to abnormal craniofacial growth patterns. These characteristics can be described as an increase in the total height of the anterior face, primarily due to a more vertical development of the lower anterior face, an increase in the mandibular plane and gonial angles as well as tilting of the palate, and a decrease in facial prognathism. The typical appearance of “adenoid facies” is also notable [11,12]. These variations in airway dimensions, along with other associated structural complexities, may contribute to the development of respiratory issues in affected individuals. Thus, early identification and appropriate intervention strategies are pivotal for effectively managing such cases [13,14,15].

We hypothesized that exploring the potential correlations between different skeletal patterns and variations in pharyngeal airway dimensions among Emirati individuals would contribute significantly to the understanding of craniofacial morphology in this specific demographic. Our findings revealed notable distinctions in pharyngeal airway dimensions across the three skeletal patterns, suggesting a potential association between the skeletal classifications and airway morphology. Recognizing these differences is essential for guiding orthodontic treatment strategies and emphasizes the necessity of integrating airway measurements into conventional orthodontic evaluations.

Many orthodontists now routinely incorporate airway assessments into comprehensive orthodontic evaluations to identify patients at risk for airway-related disorders. By addressing underlying airway concerns alongside dental alignment, orthodontic treatment can optimize respiratory functions and promote overall health and well-being for patients [11,15]. Advancements in imaging modalities, such as CBCT, have revolutionized the assessment of the pharyngeal airway in orthodontic practice. The enhanced diagnostic capabilities provided by CBCT enable precise 3D visualization of the airway and reliable dimensional measurements, facilitating informed treatment decisions [16,17]. The aim of the current study was to evaluate the pharyngeal airway dimensions of individuals with different skeletal classifications in a cohort of the Emirati population using CBCT technology.

Previous studies have demonstrated variability in the morphological aspects of the upper airway among patients with different skeletal profiles [18,19,20]. There exists a clear association between airway dimensions and skeletal growth patterns. Individuals with retrognathic mandibles in Class II occlusion typically exhibit smaller oropharyngeal dimensions, while those with prognathic mandibles in Class III occlusion tend to have larger dimensions. Furthermore, distinct airway orientations have been observed in these skeletal deformities, with Class III often characterized by a vertical airway orientation, in contrast to the forward orientation seen in Class II skeletal defects [18]. In the current study, significant differences were observed in the nasopharynx volumes among the different skeletal patterns, with skeletal Class III group participants displaying lower volumes compared to those in the skeletal Class I and II groups. This finding confirms that the severity of the skeletal pattern influences nasopharyngeal morphology, potentially impacting respiratory function and overall airway health. Furthermore, the significant positive association between anterior facial height and nasopharynx volume highlights the role of craniofacial dimensions in shaping pharyngeal airway anatomy. These findings are consistent with prior studies showing positive correlations between nasopharyngeal widths and vertical facial patterns [19,20].

In line with previous research, our study considered sex as a covariate due to documented structural and functional variations in the upper airway between men and women [21,22]. The analysis of the nasal cavity dimensions in the studied sample revealed a trend towards slightly higher mean volumes in men compared to women, although this disparity did not attain statistical significance. While the data indicated that sex did not considerably influence oropharynx or hypopharynx dimensions, it is noteworthy that we observed a very slight elevation in mean hypopharyngeal volume among women, which could possibly have been influenced by the larger female population size in our sample. In addition, there were slightly higher oropharyngeal volumes in men, consistent with previous reports [23,24].

The analysis of the MCA in the studied sample provided further insights into potential differences based on sex and skeletal classifications. Despite a slight sex difference observed in MCA volumes, with women exhibiting slightly larger mean volumes compared to men, this disparity was statistically non-significant, suggesting that sex may not be a significant factor influencing MCA dimensions. However, when considering different skeletal patterns, notable variations were observed, since the skeletal Class II group displayed the smallest MCA volume, indicating a potentially narrower airway constriction compared to the that in the skeletal Class I and Class III groups. Moreover, the distribution of MCA across different anatomical levels provides additional insights into the specific regions of airway constriction within each skeletal pattern, with the skeletal Class II group showing a propensity towards constriction in the CV2 region, while the skeletal Class III group exhibited a higher frequency of MCA occurrence at the basion and CV3 levels. These findings underscore the complexity of airway dimensions in individuals with different skeletal patterns, highlighting the importance of considering factors such as sex and skeletal classification in assessing airway function and morphology.

Overall, the interpretation of these results highlights the complex interplay of various factors, including sex, skeletal classification, and craniofacial morphology, in shaping pharyngeal airway dimensions. These findings contribute to our understanding of airway anatomy in individuals with diverse skeletal patterns and underscore the need for further research to elucidate the underlying mechanisms driving these associations. Additionally, the relatively low coefficients of determination in the regression analyses suggest that additional variables not considered in this study may influence airway dimensions.

It is well known that craniofacial abnormalities have a close association with the narrowing of the upper airway and OSA. Morbid obesity, increased body mass index (BMI), genetics, smoking, menopause, alcohol use before sleep, and night-time nasal congestion have all been considered risk factors for OSA. The treatment options for OSA range from general measures such as weight loss, avoidance of sleep in the supine position, and nasal continuous positive airway pressure (CPAP) to more invasive surgical approaches or the use of oral appliances. Oral appliances could be considered a potentially useful option, especially for patients with mild-to-moderate disease and patients who do not tolerate CPAP machines [11,25,26].

The limitations of the current study include the relatively small sample size and single-center design, which limit the generalizability of the results to broader populations. The use of CBCT imaging may introduce inherent limitations, such as the inability to capture dynamic airway changes during respiration and potential distortion artifacts affecting measurement accuracy [27]. Moreover, the exclusion of certain variables, such as soft tissue structures, airway patency, and genetic factors, from the regression models may have underestimated their impact on airway dimensions [28].

## 5. Conclusions

No significant differences were found between the airway segments and the most constricted airway area among different skeletal patterns, except for the nasopharynx volume, which was larger in the skeletal Class I group than in the skeletal Class II and III groups.

Positive correlations were found between the anterior facial height and each of the nasal cavity volume, nasopharynx volume, and total airway area.

The ANB angle had a positive correlation with the nasopharynx volume. However, the correlations with the oropharynx and the most constricted airway area were negative.

This CBCT study involved a comparative assessment of pharyngeal airway dimensions in different skeletal patterns among Emirati subjects, and the findings have implications for clinical practice and future research. Enhancing our understanding of the relationship between skeletal patterns and pharyngeal airway dimensions among Emirati patients might help guide personalized treatment approaches aimed at optimizing respiratory function in these patients.

## Figures and Tables

**Table 1:** Definitions of the airway parameters and cephalometric measurements used in this study.

Nasal cavity	The nasal cavity lies above the bone that forms the roof of the mouth and curves down at the back to join the throat (Figure 2).
Nasopharynx	The nasopharynx is the superior portion of the pharynx that is located posterior to the nasal cavity and above the soft palate (Figure 3).
Oropharynx	The oropharynx is located below the nasopharynx and can be divided into the retropalatal and retroglossal oropharynx, extending to the epiglottis (Figure 4).
Hypopharynx	The hypopharynx or laryngopharynx is that area of the pharynx caudal to the epiglottis and extending to the larynx (Figure 5).
Total airway	The airway area that includes the nasopharynx, oropharynx, and hypopharynx (Figure 6).
Most constricted area	The cross-sectional area that shows the minimum circumference of the airway border (Figure 7).
ANB	The ANB angle relates the maxillary and mandibular skeletal bases to each other.
Overjet	The horizontal distance of the maxillary incisal tip to the mandibular incisal tip.
Anterior facial height	The vertical distance from nasion to menton.

**Table 2:** The boundaries of the measured airway segments and the most constricted area of the airway.

Airway Segment	Plane	Border	Boundaries
Nasal cavity	Sagittal plane	Anterior	A line connecting the anterior nasal spine (ANS)–the tip of the nasal bone–nasion (N).
		Posterior	A line extending from sella point (S)–posterior nasal spine (PNS).
		Superior	A line connecting the N–S.
		Inferior	A line extending from ANS to PNS.
	Coronal plane		The outline of the nasal cavity in a section including the maxillary first molar bifurcation area starting at the crista galli, running downward toward the nasal floor and passing through the sidewalls of the right and left nasal cavity.
Nasopharynx	Sagittal plane	Anterior	A line extending from S to PNS.
		Posterior	A line extending from S to tip of the odontoid process.
		Inferior	A line extending from PNS to tip of the odontoid process.
Oropharynx	Sagittal plane	Anterior	A line extending from PNS to menton.
		Posterior	A line extending from the tip of the odontoid process to posterior–superior border of CV3.
		Superior	A line extending from PNS to tip of the odontoid process.
		Inferior	A line extending from the anterior–superior border of CV3–menton.
Hypopharynx	Sagittal plane	Posterior	A line extending from the anterior–superior border of CV3 to anterior superior border of CV4.
		Superior	A line extending from menton to anterior–superior border of CV3.
		Inferior	A line extending from the posterior–inferior corner of CV4 to menton.
Total airway	Sagittal plane	Anterior	A line extending from S to menton.
		Posterior	A line extending from S to anterior superior border of CV4.
		Inferior	A line extending from the anterior superior border of CV4 to menton.
Most constricted area	Sagittal plane		The cross-sectional area that shows the minimum circumference of the airway border along the total airway area that includes nasopharynx, oropharynx, and hypopharynx.

**Table 3:** Descriptive statistics of the airway and cephalometric parameters.

Parameters	n	Min	Max	Mean (SD)
Nasal cavity	103	16465	45932	28340.9 (6207.47)
Nasopharynx	103	1964.0	14,393.0	6730.7 (2270.30)
Oropharynx	103	3489.0	29,789.0	14,045.8 (5850.44)
Hypopharynx	103	1545.0	31,748.0	4534.8 (3132.38)
Total airway	103	9001.0	48,227.0	24,702.5 (8344.49)
MCA	103	31.0	517.0	209.5 (106.40)
Overjet	103	−4.7	12.3	2.8 (2.03)
ANB angle	103	−10.3	10.0	3.6 (2.74)
Anterior facial height	103	102.1	125.1	114.6 (5.24)
Age	103	19.0	68.0	34.2 (11.43)

**Table 4:** Correlation coefficients between the airway parameters and cephalometric parameters in all included subjects.

	Nasal Cavity	Nasopharynx	Oropharynx	Hypopharynx	Total Airway	Most Constricted Airway Area	Overjet	ANB	Anterior Facial Height	Age
Nasal cavity	1.000	0.367 **	0.128	−0.092	0.190	−0.053	−0.011	0.141	0.270 **	0.098
Nasopharynx		1.000	0.425 **	0.163	0.626 **	0.308 **	0.131	0.206 *	0.306 **	0.074
Oropharynx			1.000	0.441 **	0.958 **	0.851 **	−0.206 *	−0.204 *	0.178	−0.246 *
Hypopharynx				1.000	0.454 **	0.534 **	−0.039	−0.100	0.025	−0.074
Total airway					1.000	0.841 **	−0.122	−0.140	0.240 *	−0.187
MCA						1.000	−0.190	−0.220 *	0.040	−0.264 **
Overjet							1.000	0.602 **	0.134	0.233 *
ANB angle								1.000	0.283 **	0.065
Anterior facial height									1.000	0.518
Age										1.000

** Correlation is significant at the 0.01 level (2-tailed). * Correlation is significant at the 0.05 level (2-tailed).

**Table 5:** Comparison of the airway parameters among all groups.

Parameters	Group 1: Class I	Group 2: Class II	Group 3: Class III	*p*-Value
Nasal AV	Mean (SD)	Mean (SD)	Mean (SD)	
Nasal cavity	2874.34 (6372.76)	5896.37 (5896.37)	26681.73 (6574.50)	0.370
Nasopharynx	7175.94 (2294.27)	6900.80 (2274.1)	5667.00 (2270.3)	0.031 *
Oropharynx	15,065.77 (5428.66)	13,002.13 (5634.27)	14,605.32 (6786.89)	0.132
Hypopharynx	5153.20 (4907.12)	4031.59 (1400.66)	4603.41 (1808.36)	0.349
Total airway	26,299.51 (8612.76)	23,524.00 (7816.98)	24,626.36 (8936.81)	0.336
MCA	220.51 (100.01)	191.93 (103.45)	228.77 (120.80)	0.296

* Significant at *p* ≤ 0.05.

**Table 6:** The location of the most constricted airway area among the groups.

Airway Level		Class I (n = 34)	Class II (n = 47)	Class III (n = 22)
Level 1 (n = 8)	Basion	2	2	4
Level 1 (n = 74)	CV2	25	35	14
Level 1 (n = 20)	CV3	6	10	4
Level 1 (n = 1)	Oropharynx	1	0	0

## Data Availability

The data presented in this study are available upon request from the corresponding author.
